# Brain aromatase modulates cardiac functions in embryonic zebrafish

**DOI:** 10.1080/23144599.2019.1675287

**Published:** 2019-10-18

**Authors:** Zulvikar Syambani Ulhaq

**Affiliations:** Department of Biomedical Science, Faculty of Medicine and Health Sciences, Maulana Malik Ibrahim Islamic State University of Malang, Batu, Indonesia

**Keywords:** Brain aromatase, oestradiol, cardiovascular, zebrafish

## Abstract

Oestradiol (E_2_) is known as a female reproductive hormone with pleiotropic effects on the cardiovascular system. Local E_2_ biosynthesis such as in the brain and myocardial cells have important physiological and pathophysiological roles. E_2_ production is catalysed by aromatase (Aro) enzyme. In teleost, two Aro isoforms are distinctly expressed in the ovary and brain. In this study, the role of brain Aro (AroB) in modulating cardiovascular system is investigated. AroB MO-mediated knockdown decreased ventricular functions. Moreover, embryos injected with AroB MO displays a sign in developing heart failure. All the effects caused by AroB MO were partially reversed by exposure to E_2_. Taken together, this study demonstrates the role of AroB in modulating normal cardiovascular function in zebrafish embryos.

## 1. Introduction

E_2_ is synthesized from testosterone by the action of Aro enzyme [1]. Interestingly, zebrafish having two Aro isoforms, ovarian Aro (AroA) and brain Aro (AroB), encoded by *cyp19a1a* and *cyp19a1b*, respectively [2]. Previously, it has been reported that knocking down of AroB decreased the heart rate (HR) in zebrafish [3], indicating that oestradiol produced in the brain regulates the heart and circulatory physiology during development. In addition, zebrafish exposed to Aro inhibitor (AI) affect embryonic heart and function [4]. Indeed, Aro is expressed in the heart tissues in mice [5]. Though there is a variation of Aro isoforms expressed in teleost fish heart [3], together these reports suggest a prominent role of E_2_ in the cardiovascular system.

Oestrogen signalling pathways are mainly through the binding of E_2_ to the oestrogen receptor (ER) that plays a critical role during sex differentiation, brain development and cardiovascular physiology [3,6]. ERα has been reported predominantly expressed in rat ventricular myocytes and zebrafish heart [7,8]. Furthermore, treatment of E_2_ and ERα attenuated isoproterenol-induced cardiac hypertrophy in H9c2 cells [9], indicating beneficial action of E_2_-induced cardioprotection. Moreover, overexpression of Aro improved functional recovery post-ischaemia in mice [10]. Although gonadal tissue is the main site for E_2_ synthesis, locally E_2_ production in extragonadal tissues such as heart and brain likely has a specific role on these organs. Therefore, this study helps to reveal the possible functions of E_2_ produced by AroB in modulating normal heart development and function in embryonic zebrafish.

## 2. Materials and methods

### 2.1. Fish maintenance and embryo culture

Adult zebrafish (*Danio rerio*) purchased from a local pet store were raised in a 60-litre tank at 26–30°C under the 14 h dark/10 h light cycle. Fertilized eggs were rinsed in embryo medium (EM) (0.004% CaCl_2_, 0.163% MgSO_4_, 0.1% NaCl and 0.003% KCl) and cultured in a six-well plate (30 eggs/8 mL EM/well) at 28 ± 0.5°C. All experimental procedures and maintenance of fish were conducted in accordance with the Guide for Care and Use of Laboratory Animals published by the US National Institutes of Health [11].

### 2.2. Exposure experiment

17β-oestradiol (E_2_, Sigma) was dissolved in dimethyl sulphoxide (DMSO) and diluted in EM at the concentrations indicated in the experiments. The final concentration of DMSO was 0.1%, which was also used for vehicle control. Exposure started at 2 hpf and the media were changed daily.

### 2.3. Morpholino-mediated knockdown of AroB

Morpholino antisense oligonucleotides (MO) were purchased from Gene Tools (USA). The sequence of AroB MO designed to block translation of *cyp19a1b* mRNA and its inverted sequence for injection control (InvB MO) were used in the previous study [3]. The efficacy of AroB MO-mediated knockdown was previously evaluated [3].

### 2.4. Measurement of cardiac functions

Embryos at 48 hpf were mounted in 3% Methyl Cellulose and the videos were recorded under a light microscope (Olympus CX21) for 1 min. Cardiac functions then were measured as previously described [12–14]. Eight to ten embryos per group were examined, and the experiments were repeated three times with eggs collected from different spawns.

### 2.5. Statistical analysis

Data were presented as mean ± SEM. Statistical differences between groups were evaluated by one-way ANOVA followed by the least significant difference (LSD) post hoc test using StatPlus. Significant differences were accepted when *p* < 0.05.

## 3. Results

**Figure 1.**
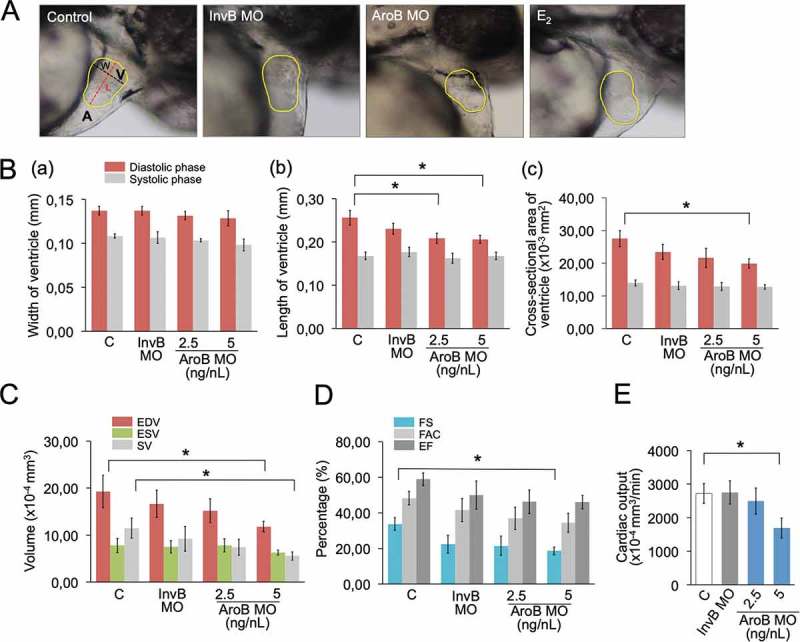
AroB MO-mediated effects on cardiac functions. (a) Representative images of a lateral view of heart region of 48-hpf embryos (uninjected control (C), InvB MO-injected and AroB MO-injected embryos, and E_2_ exposed embryos). (a) Atrium; V, Ventricle; L, Length; W, Width. (b) Measurement of heart ventricle at diastolic and systolic phase. Width of ventricle (a); length of ventricle (b); cross-sectional area of ventricle (c). (c) Measurement of EDV, ESV and SV. (d) Measurement of FS, FAC and EF. (e) Measurement of CO. Data are presented as a mean ± SEM. (*) indicates a significant difference compared to control (*p* < 0.05). Ns, not significant; EDV, end-diastolic volume; ESV, end-systolic volume; SV, stroke volume; FS, fractional shortening; FAC, fractional area change; EF, ejection fraction; and CO, cardiac output.

To determine whether AroB plays an important role in cardiac functions, MO experiment was performed. AroB MO used in this experiment is characterized from a previous study [3]. There is no off-target effect caused by AroB MO, and the injection of 5 ng/nL AroB MO able to inhibit the translation of AroB [3], indicating the specificity of AroB MO. Heart ventricle size was evaluated by measuring the width, length and cross-sectional of ventricle area during diastolic and systolic phase (Figure 1(a)). When AroB MO was injected, there were no significant changes in the width of ventricle during diastolic or systolic phase (Figure 1(b)-a). Length of the ventricle was significantly decreased in 2.5 ng/nL and 5 ng/nL groups, while cross-sectional area of the ventricle was significantly decreased only in 5 ng/nL group compared to uninjected control during diastolic phase, but not systolic phase (Figure 1(b,c)). Moreover, embryos injected with 5 ng/nL AroB MO showed a significant decreased on end-diastolic volume (EDV), stroke volume (SV), fractional shortening (FS) and cardiac output (CO) compared to uninjected control, but not end-systolic volume (ESV), fractional area change (FAC) and ejection fraction (EF) (Figure 1(c,e)). Therefore, embryos injected with 5 ng/nL AroB MO were used for further experiments. Injections of InvB MO did not show any significant difference in all parameters measured compared to the uninjected control (Figure 1(b–e)). In addition, there is no change on body length of zebrafish embryos injected with AroB MO or InvB MO compared to control (data not shown), indicating that AroB modulated the changes on ventricular size and cardiac functions.

**Figure 2.**
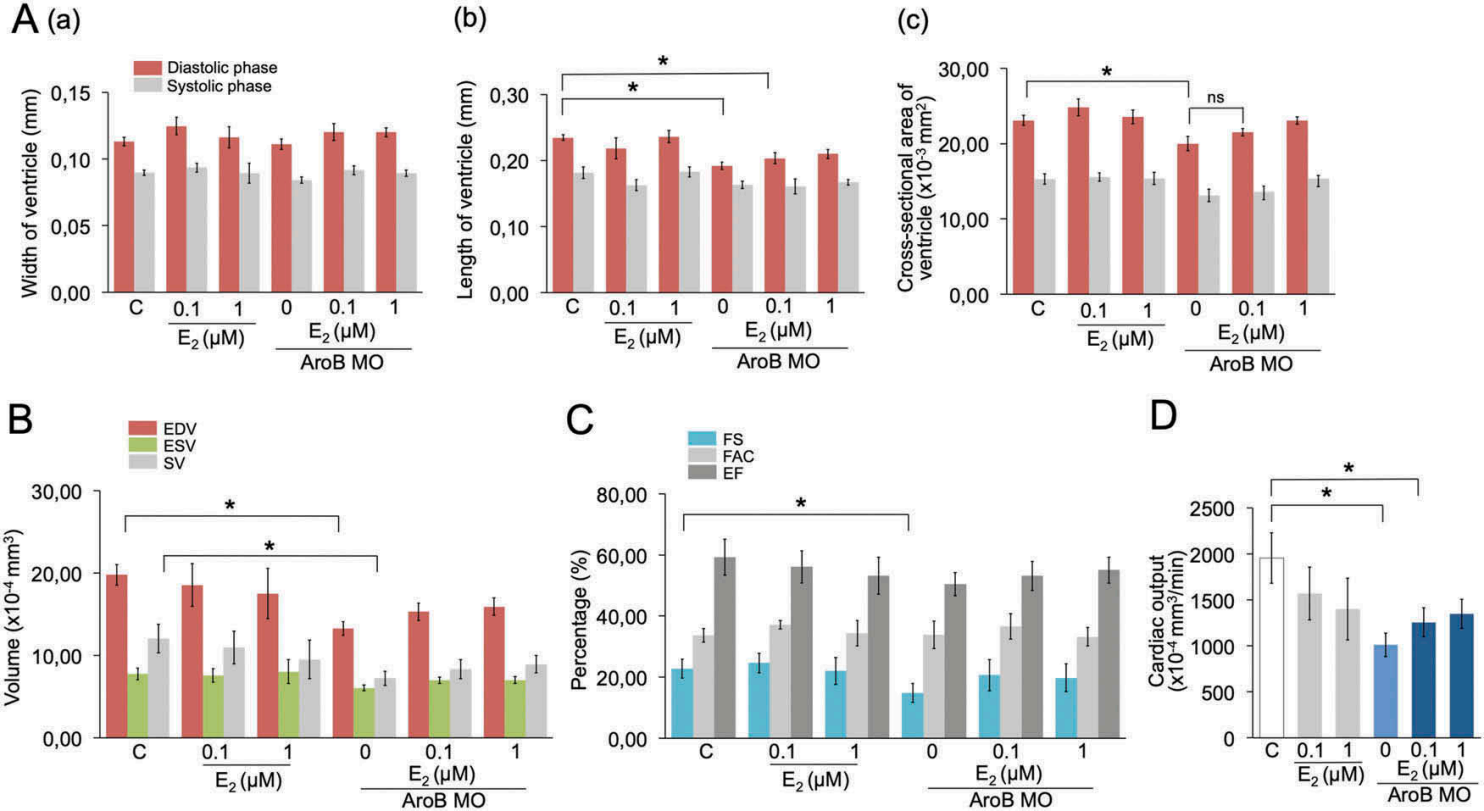
E_2_ reversed the effects of AroB MO-mediated knockdown on cardiac functions. (a) Measurement of heart ventricle at diastolic and systolic phase. Width of ventricle (a); length of ventricle (b); cross-sectional area of ventricle (c). (b) Measurement of EDV, ESV and SV. (c) Measurement of FS, FAC and EF. (d) Measurement of CO. Data are presented as a mean ± SEM. (*) indicates a significant difference compared to control (*p* < 0.05).

Because AroB MO inhibits the synthesis of endogenous E_2_, AroB MO-injected embryos were exposed to E_2_ to investigate whether E_2_ able to ameliorate the effects caused by AroB MO. In this experiment, the dose of E_2_ was applied according to a previous report [3]. Besides, embryos exposed to E_2_ range from 0.01–1 μM able to induce AroB expression without showing the developmental defects [2,15]. Exposure to E_2_ did not show any changes on morphology of the ventricle (Figure 1(a)) and in all parameters measured (Figure 2(a–e)). Exposure to 1 μM E_2_ partially reversed all the parameters caused by AroB MO (Figure 2(b–e)).

## 4. Discussion

This report demonstrated that heart development and functions were affected by AroB MO-mediated knockdown. Moreover, E_2_ attenuated the effects caused by AroB MO, suggesting the necessity of E_2_ produced by AroB in the cardiovascular system. The decreased of ventricular size during diastolic phase, EDV, FS, SV and CO with normal EF in AroB MO-mediated knockdown showed similar features as observed in patients with heart failure with preserved ejection fraction (HFpEF), resulted from left ventricular diastolic dysfunction (LVDD) [16]. Previous study indicates that Aro deficient (ArKO) mice result in a significant decrease in ventricular weight index (VWI), although no changes in total heart weight [17]. Similarly, zebrafish embryos exposed to AI shows smaller ventricular area [4], which is support the possibility that AroB is involved in modulating cardiotropic effect.

Ventricular dysfunction in MO-mediated knockdown could be a result of ventricular tissues remodelling. E_2_ deficiency is known to stimulate cardiac inflammation and subsequently promote myocardial fibrosis and hypertrophy [16]. The action of E_2_ through ERα mediates the anti hypertrophic effect induced by angiotensin II [18]. In addition, GPR30 KO mice shows impairment in left ventricular cardiac functions [19], indicating both genomic and non-genomic E_2_ signalling pathways are involved in the development of ventricular dysfunction. However, histological tissue changes in MO-mediated knockdown need to be verified.

Previously, it is reported that AroA but not AroB is expressed in adult zebrafish heart, and E_2_ produced in the brain control the heart rate through central serotonin (5-HT) signalling [3]. Both expressions of tryptophan hydroxylase isoforms (TPH1, TPH2), a rate-limiting enzyme for 5-HT production, are lower in embryos exposed to E_2_ [3], suggesting that both central and peripheral 5-HT are under the control of E_2_ signalling. Both TPH isoforms are expressed in human [20] and zebrafish heart (data not shown), giving a possibility that MO-mediated knockdown may directly affect TPH expressed in cardiac tissues to regulate the 5-HT level and cardiac function. Indeed, brain 5-HT dysfunction plays a critical role in the pathophysiology of HF [21]. Furthermore, disruptions of non-neuronal TPH1 in mice lead to HF without affecting structural defect [22]. Therefore, further experiment needs to be done to understand the role of E_2_ on 5-HT neuron in zebrafish heart.

## 5. Conclusion

In conclusion, this study provides evidence that E_2_ produced by AroB plays a significant role in normal heart development and functions in embryonic zebrafish.
